# Evolving trends in cryptomining malware: a systematic literature review

**DOI:** 10.1186/s40163-025-00267-5

**Published:** 2026-01-31

**Authors:** Ema Mauko, Sara Rubini, Chimdi Igwe

**Affiliations:** 1 Department of Security and Crime Science, University College London, 35 Tavistock Square, London, WC1H 9EZ UK; 2 Department of Computer Science, University College London, 66 - 72 Gower Street, London, WC1E 6EA UK

**Keywords:** Systematic review, Cryptomining malware, Cryptojacking, Cryptocurrency, Cybercrime

## Abstract

The rise of *‘cryptojacking’* – the covert use of victim resources for unauthorised cryptocurrency mining – has become a significant cyber threat since the introduction of Bitcoin. Such cryptomining malware secretly hijacks a user’s computational power to generate cryptocurrency without their knowledge or consent, leading to reduced and/or degraded performance at the victim’s expense. This paper presents a systematic literature review of 119 articles tracing the evolution of cryptomining malware, past trends in their dissemination and detection, security recommendations, and anticipated future developments. We specifically highlight the dual impact of this threat, which targets not only individual users on devices like IoT, mobile phones, and cloud infrastructures, but also critical national infrastructure, large corporate networks, and high-traffic websites. Our analysis reveals that the threat landscape, which significantly expanded around 2017, continues to grow steadily. Additionally, we systematically identify and discuss detection methods – such as network traffic analysis, CPU utilization monitoring, and machine learning classifiers – as well as security recommendations like browser extensions, patch management, and network-level blocking. Our findings highlight the urgent need for a unified, multi-stakeholder security strategy to mitigate this pervasive and adaptable threat.

## Introduction

Cryptocurrencies are media of exchange which – as opposed to traditional fiat money – instead use blockchain technology and cryptography to regulate the release of new monetary units and verify transactions (Pastor et al., 2020). Money can be transferred from user to user by committing a transaction record to a distributed write-only ledger called the *blockchain*. In the blockchain, information - in this case transaction records - is stored in *‘blocks’* which are cryptographically linked into a chain, thus making up a ledger that is distributed across a network. This blockchain is then maintained by a network of *‘miners’* that validate new blocks and are rewarded with cryptocurrencies for doing so (Konoth et al., 2018). This process is of generating new cryptocurrency units is therefore called *cryptocurrency mining* (*‘cryptomining’*). While different cryptocurrencies incorporate different mechanisms for validating transactions, one of the most common mechanisms is the *Proof of Work* (POW) mechanism, which involves solving a highly complex cryptographic problem. Initially, the cryptographic puzzles involved in transaction validation required specialist, often expensive hardware to solve (Aljehani & Alsuwat, 2022); while the original Bitcoin proof-of-work puzzle was simply a time-consuming hashing exercise (SHA-256) designed to be cheap and viable on CPUs, its nature meant that it could be performed much more efficiently on GPUs and specialized hardware. This difference in efficiency led to a high barrier to entry as mining on general-purpose CPUs became economically unviable. However, in recent years the barrier of entry has been lowered with the advent of CPU-based mining algorithms introduced by the Monero cryptocurrency (Seigen & Jameson, 2013) which were specifically designed to resist acceleration by more specialized hardware.

A distinguishing feature of cryptocurrencies is the pseudo-anonymity of the transactions. For this reason, since the introduction of Bitcoin in 2009, cybercriminals have largely opted to use cryptocurrencies to perform and receive payments. The financial reward offered at the end of transaction validation has also led to the development of a new form of malware, known as *cryptomining malware*. This form of malware exploits the user’s computational resources without their knowledge or consent with the purpose of obtaining cryptocurrencies (Gomes and Correia, 2020). Initially, there were two main types of cryptomining malware. The first is host-based cryptojacking, which is a malicious application that is launched on an affected computer, much like a computer virus. The second and most common form is *browser-based cryptojacking*, which occurs when a user visits an infected website and cryptomining code is executed in the background (Tanana, 2020).

While the literature often references both *‘cryptojacking’* and *‘cryptomining malware’* interchangeably, we will firstly clarify the distinction between the two. *‘Cryptojacking’* is a form of ‘illegal cryptomining’, which refers to the illegal act described above. The various vectors through which such acts are achieved are known – individually and collectively – as *‘cryptomining malware’*. Hence, this work will utilise these terms accordingly.

Cryptomining malware is not a new phenomenon. Since the introduction of Bitcoin, users have begun to develop malware for the sole purpose to mine cryptocurrencies, and with the surge of value in cryptocurrencies the cyber security landscape has been drastically transformed. Beginning from browser-based cryptojacking, attack methods have evolved to include IoT, cloud and mobile-based cryptomining malware. In this complex scenario, end-users, and enterprises represent appealing targets alike. Data shows that in 2018, cryptomining malware affected 42% of organizations worldwide (Cybersecurity Industry, 2018), and in 2021 the European Union Agency for Cybersecurity listes cryptojacking as the third most common cybersecuirty threat (Sriman et al. 2023). Indeed, due to companies’ extensive resources, attacks on enterprises have increasingly become an appealing economical target for criminals, who have three options to use the money (Goyal and Matta 2023):

1. Use exchanges or peer-to-peer transactions to convert it to fiat cash;
2. Use it as a service cryptocurrency;
3. Employ services for blending cryptocurrencies to cover its trail.

The increasing profitability of cryptomining malware stems from a confluence of rising cryptocurrency values, growing computational power availability, and escalating electricity costs - all of which create a lucrative opportunity for cybercriminals to exploit compromised systems as passive income sources. The value of Bitcoin alone has surged by over 540% between 2020 and 2024, directly impacting the potential revenue from cryptomining malware infections (Pastrana & Suarez-Tangil, 2019). Furthermore, mining difficulty and required computational power have increased significantly, incentivising attackers to hijack distributed resources to reduce their own infrastructure costs (Gaies et al., 2025). With global electricity prices also rising, particularly in regions with deregulated markets, malicious actors increasingly transfer the financial burden to unsuspecting users, maximising profits while avoiding direct costs (Hajiaghapour-Moghimi et al., 2024).

At present – to the best of our knowledge – no systematic review has investigated past, future trends, proposed detection and mitigation techniques of cryptomining malware. As such, the aforementioned convergence of economic incentives, and its impact on the rapid evolution in cryptomining attack techniques and ecosystem trends, offers a critical gap in the crime science and policy literature for a systematic methodology to comprehensively assess evolving attack vectors and defence mechanisms. A systematic literature review serves as an essential methodology for a robust overview of the previous work done on the topic thus enabling us to synthesize dispersed knowledge, identify evolving attack vectors, and forecast technological adaptations by adversaries based on emerging trends and techniques (Cletus et al., 2024). By examining past incidents and present detection frameworks, systematic reviews also illuminate how current solutions align with predicted developments in malware sophistication and attack surfaces. This panoramic understanding not only supports robust knowledge consolidation but also empowers the cybersecurity community to anticipate and strategically counter the evolving threat of cryptojacking.

The remainder of the paper is organised into four main sections. The methodology and search strategy undertaken in this study is explained in Sect. 2. Subsequently, the results of the review are presented in Sect. 3 and further discussed in Sect. 4. Lastly, we conclude the review in Sect. 5.

## Method

### Systematic review

Prior to conducting this study, a review protocol was generated which went through three rounds of internal feedback with subject experts within our departments. The protocol was then adjusted based on the feedback received, and the final review protocol (see Appendix A). Furthermore, the researchers followed the PRISMA methodology to ensure the validity of the systematic review (PRISMA Statement, 2025). The research questions of this systematic review are as follows:

**RQ1:** *What are the latest trends in cryptomining malware?*

**RQ2:** *How are these trends expected to evolve in the next few years?*

**RQ3:** *What security recommendations have been suggested to mitigate cryptomining malware?*

### Inclusion criteria

The inclusion criteria for academic and grey literature remained consistent in order to be considered for review:

- Any type of study design: Qualitative and quantitative including systematic reviews and meta-analyses; experimental studies; case reports.
- Discusses malware that specifically exploits computational resources for cryptomining purposes.
- Published between 2018 and 2023 (inclusive).
- Available in English.

In addition to these criteria, for grey literature specifically, we included industry reports, government and official publications and commercial reports. This inclusion criterion was applied to search the following academic databases: IEEE Xplore; Web of Science; ProQuest; and ACM Digital Library; as well as Policy Commons for grey literature. To ensure other relevant academic articles not covered within the journals searched by these databases (such as conference proceedings and preprints) Google Scholar was approached as a final step search – however, the search strategy was modified to only include peer-reviewed publications with titles including the search terms, given the limitations of the database.

The six-year time frame (2018–2023) for the literature search was chosen for two main reasons. Firstly, preliminary searches suggest increased (academic) interest in browser-based cryptomining (and cryptocurrency mining at large) from around 2018. This timing aligns with the lowered barrier of entry for malicious activity due to the proliferation of CPU-based mining, which began allowing attackers to profitably hijack standard consumer devices (starting prominently around 2017). This technological and criminological shift justifies our selected 2018–2023 review period, capturing the years immediately following the emergence of widespread cryptojacking. Secondly, the selected time period encapsulates the period before, during and after the COVID-19 pandemic – the impact of which on cyber-criminal activity has well noted in other work (Buil-Gil et al., 2021; Hoeboer et al., 2024; van de Weijer et al., 2024). As such, the selection allows for a relative comparison of the pre- and post-pandemic landscape *vis-à-vis* cryptomining malware.

### Search strategies

#### Academic databases

For all academic databases, we used the same search string given below. The search string covers popular alternative terms used to refer to cryptomining such as *“cryptojacking”* (lines 1 and 2) and requires the article to talk about malware specifically rather than legitimate uses of cryptomining (line 3).

1 “crypto*”

 2 AND (“mining” OR “mine*” OR “jacking”)

3 AND (“malware” OR “malicious software” OR “attack*”)

#### Grey literature

For grey literature, the following search string was used for the Policy Commons database.

1 cryptomining malware

2 OR cryptojacking

3 OR “crypto mining malware” 4 OR “crypto jacking”

As with the search strategy for academic databases, only reports published between 2018 and 2023 and in English were considered. The broad search terms maximised the diversity of disciplines captured within the systematic strategy, which was crucial to the interdisciplinarity required of this research question. Non-technical terminology was particularly effective for any policy related disciplines and grey literature. A pilot search protocol underwent internal peer and external stakeholder review. Crucially, it ensured a currency-agnostic approach, which aided in seeing which currencies featured most heavily in the literature.

### Selection of studies

After completing the search strategy outlined in the section above, identified references were compiled into a spreadsheet for further analysis. First, duplicate entries were identified and removed. Second, 20% of the literature abstracts were screened by the four researchers collectively against the inclusion criteria described above. In the case that the inclusion could not be determined based on the abstract alone, the full text was screened. Specifically, 5% of the abstracts were screened by all four researchers, and the remainder 15% were screened by rotating pairs of researchers (2.5% of the sample per pair before the pair is changed). This was done to establish a common and unbiased understanding of the inclusion criteria amongst the researchers and ensure the replicability of the research in general.

In order to quantify the inter-rater reliability (IRR) and evaluate the chance of agreement, the prevalence- and bias-adjusted kappa (PABAK) statistic was calculated (Chen et al., 2009). The PABAK score after screening 20% of the sample was 0.8853, indicating a high degree of agreement amongst the researchers. Following this, the rest of the literature was split equally among four researchers to review the abstracts and evaluate the inclusivity of each research paper individually. Once this was complete, the articles were screened in full and further in-depth thematic analysis commenced with a view to answer the research questions outlined above.

#### Note on exclusion rate and *‘selfish mining’*

The search terms were necessarily broad to ensure comprehensive coverage of the malware domain. However, these terms inherently overlap with articles discussing the broader protocol-level vulnerabilities, leading to a high exclusion rate for papers discussing *‘selfish mining’*, as opposed to cryptojacking or cryptomining malware. Whilst this has become a topic of interest raised by many papers, this review considers selfish mining as distinct to the issue of cryptomining malware, and as such does not fall under its purview. Our rationale for this decision is primarily due to the target (and nature) of the attack. Cryptojacking leverages unauthorised use of given computing resources for the attacker’s profit, victimising the resource owner. In contrast, *selfish mining* refers to a strategy within a mining pool, in which a miner withholds the release of already mined blocks until others have gone further in the computational chain, in an attempt to claim a larger share of the mining profits than others within the blockchain (Eyal & Sirer, 2018). This is effectively a protocol-level attack or a strategic deviation within the consensus mechanism of a cryptocurrency network (specifically Proof-of-Work systems like Bitcoin); a consequence of the blockchain’s initial dependence on an *‘honor code’* between individual miners – hence, the victim is the integrity of the blockchain network itself, as well as competing miners.

### Data extraction and management

A pro forma to capture relevant information from each article was developed and piloted. From each selected academic paper, the following information was extracted:

- Publication metadata - year of publication, country of origin (for grey literature).
- Methodology - type of study, quality of evidence (adapted from Blythe and Johnson (2021)).
- Main theme of the paper.
- Past trends identified.
- Future trends identified.
- Security recommendations identified.

## Results

### Summary of search results

**Fig. 1:**
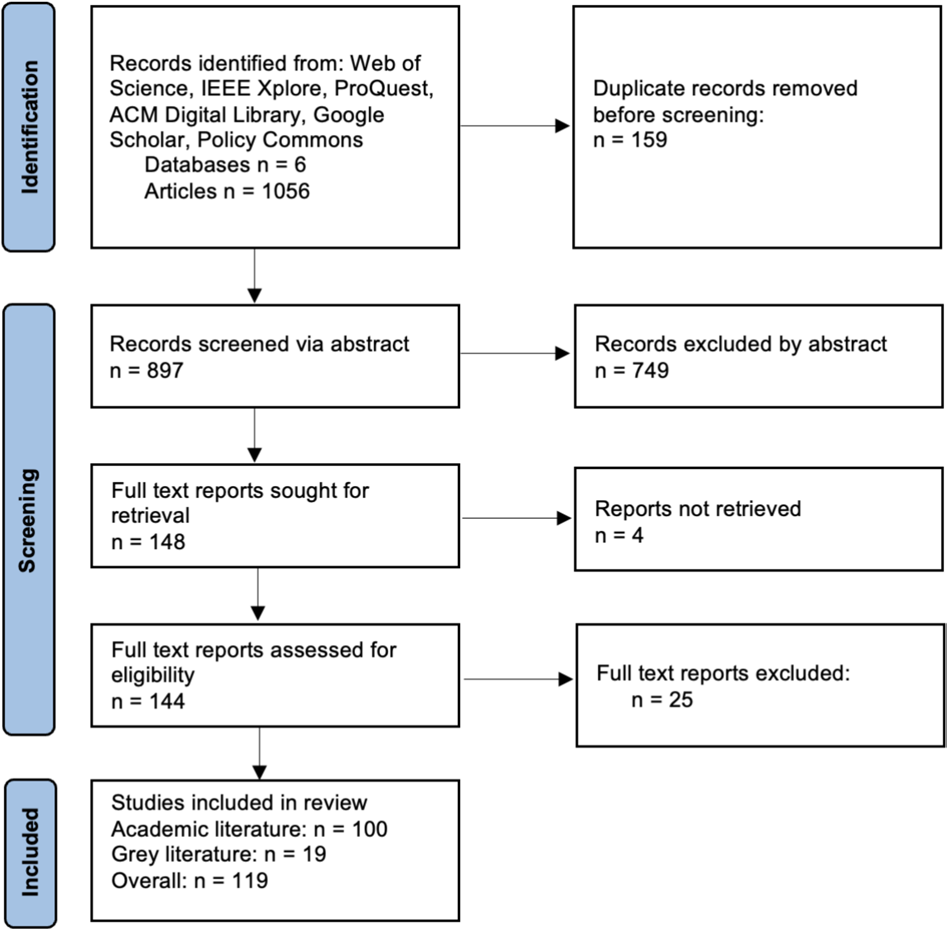
PRISMA chart summarising the attrition of articles throughout the search process

The PRISMA chart in Fig. 1 summarises the study selection process. The initial database search yielded a total of 1056 articles, with 965 from academic databases and 91 from grey literature, all which were collected and screened for eligibility. After removing duplicate entries, 897 articles remained, of which 749 articles were excluded based on their abstracts, while 148 entries were included in the full-text analysis. A large percentage of articles excluded during the screening phase was related to selfish mining, a topic beyond the scope of this review At the final stage of full-text screening, a further 29 articles were excluded due to an inability to retrieve full text articles or a lack of discussion of cryptojacking or cryptomining malware within the article. The reported findings in this section are based on 119 articles that satisfied all the criteria reported in the section above.

The synthesis of articles includes 19 articles from grey literature. These include mostly reports produced by various governmental and non-governmental entities from the United States (six), the European Union (three), Malaysia, Switzerland, Philippines, France, India, The Netherlands and the United Kingdom (two), with two further articles coming from institutions not tied to a specific region.

**Table 1:** Number of articles per year included in the research, separated by academic and grey literature

Year	Academic lit	Grey lit	Sum
2018	13	4	17
2019	22	7	29
2020	24	3	27
2021	11	1	12
2022	15	2	17
2023	16	1	17

Although the present systematic review aims to bring together a diversity of studies and methodology, it is necessary to consider how some studies provide more rigorous evidence than others. In order to assess this in a systematic way, a hierarchy of evidence was developed based on Blythe and Johnson (2021) and summarised in Table 2 . This ordering places provided evidence in terms of tangibility and reproducibility - with speculative studies at the lowest tier, whilst real-world experiments are considered the highest standard. In the following sections, we explore past and future trends, malware detection and security recommendations as they emerge in the included sources .

**Table 2:** Hierarchy of evidence used in the systematic review

Evidence type	Description	Label
Real world	Paper demonstrates an attack or consequence implemented “in the wild” on a real IT system, or data analysed in the paper is collected by the authors of the paper themselves from the wild	Real-world.
Experimental (lab based)	Paper demonstrates an attack or consequence in a lab-based experiment using physical IT systems.	Lab-based
Experimental (simulation)	Paper demonstrates an attack or consequence in a computer-generated simulation.	Simulation
Speculative	Data speculatively derived by a group of experts, authors, or users. Data may be supported by relevant case studies.	Speculative

### Past trends

**Fig. 2:**
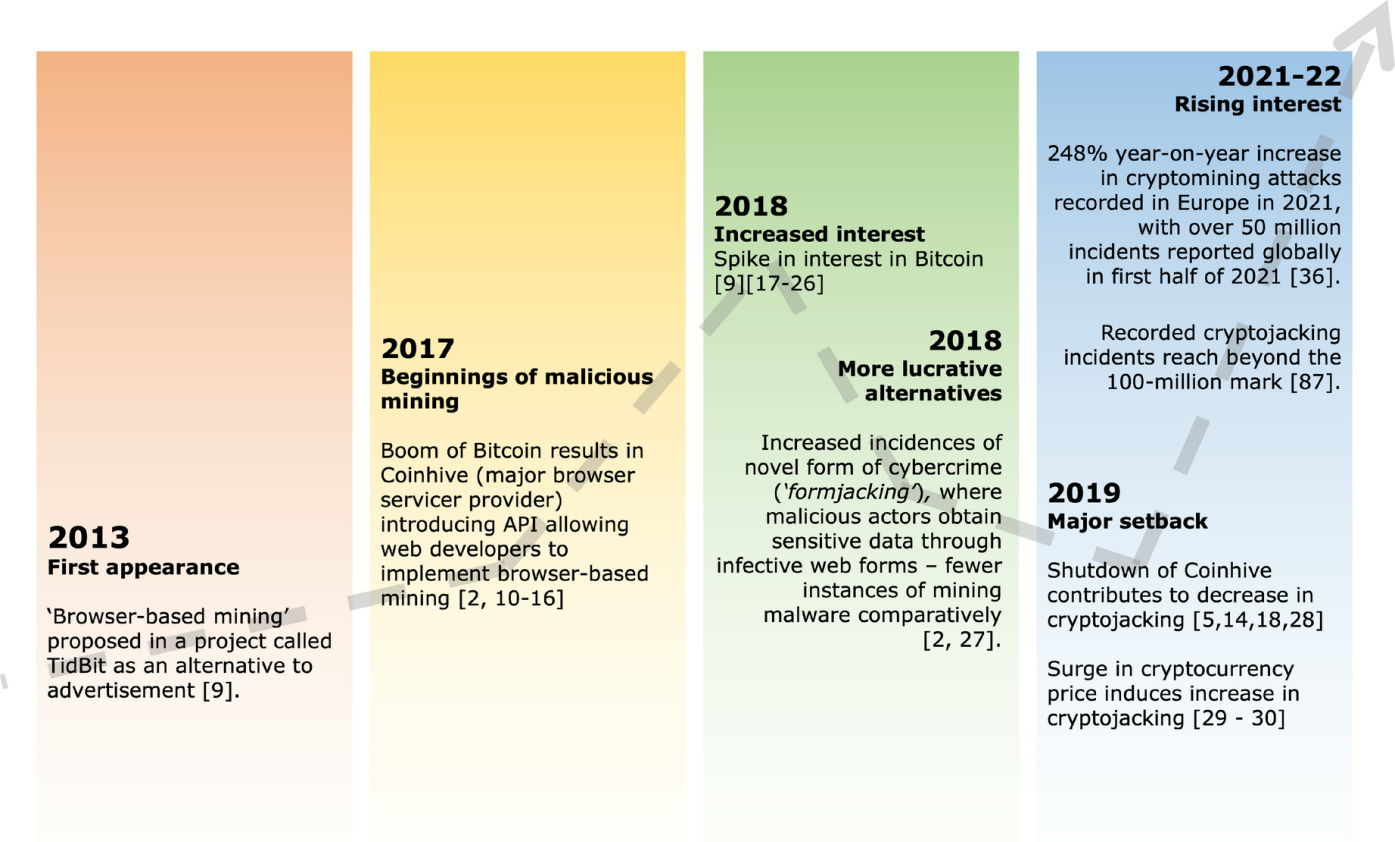
Timeline summarising the key cryptojacking past trends identified

The following section presents the past trends as reported in the literature. Figure 2 presents a visual summary of the observed trends in cryptojacking as referenced in the literature. Researchers point to a hackathon project called *TidBit*, proposed in 2013, as a precursor to modern day cryptomining malware (Tanana, 2020; Carlin et al., 2020). The initial proof-of-concept conceptualised cryptocurrency – specifically Bitcoin – mining on website users’ devices as an alternative to conventional advertising. However, most articles associate the start of browser-based cryptomining to Coinhive, a browser-based service that, in 2017, introduced an application programming interface (API) which allowed developers to implement browser mining on their website. Following Coinhive’s shutdown in 2019, fluctuating interest in cryptojacking were largely noted to oscillate in step with wider interest in cryptocurrencies.

**Fig. 3:**
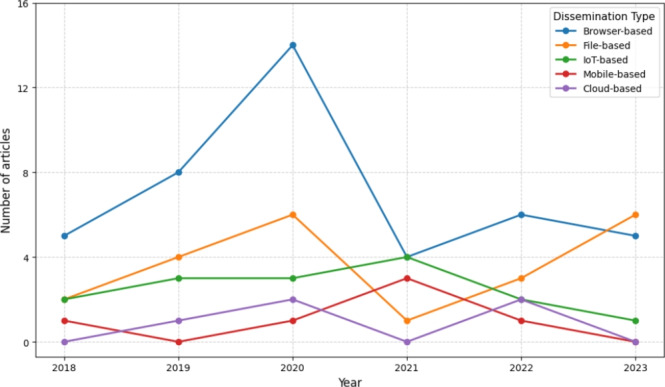
Timeline summarising the key cryptojacking past trends identified

The literature also notes a variety of cryptomining malware dissemination vectors utilised by malicious actors through a variety of vectors. The principal methods identified in the literature include: *browser-based malware*, which operates via embedded scripts on compromised or malicious websites; *mobile-based malware*, which targets smartphones and tablets to exploit their processing power; *IoT-based malware*, which infects internet-connected devices with limited security protections; *file-based malware*, typically delivered through malicious downloads or email attachments; and *cloud-based malware*, which compromises cloud infrastructure to harness substantial computational resources for illicit mining. Table 3 offers further information on the past trends in the dissemination of malware, whilst Fig. 3 offers a visual representation of the research focus of each category across each year. As can be seen in Fig. 3, browser-based dissemination has consistently attracted the most academic focus over the review period. Similarly, academic focus on all vectors received heightened attention in 2020, with 26 articles discussing cryptomining malware dissemination. From an evidentiary standpoint, the majority of these articles are categorised as *‘experimental (simulation)’* approaches – as per our hierarchy of evidence, reflecting a controlled approach to investigating cryptomining. However, around 15% of the collected articles studied cryptomining malware *‘in the wild’*, providing valuable, real-world evidence of criminal approaches.

**Table 3:** Summary of the main past trends in the dissemination of cryptomining malware, corresponding to the hierarchy of evidence in Table 2

Dissemination	Description	Real world	Lab based	Simulation	Speculative
Browser	Malicious actors deploy JavaScript scripts through malicious websites to exploit user’s computational resources without their knowledge or consent with the purpose of mining cryptocurrencies	Konoth et al. (2018), Burgess et al. (2019), Saad et al. (2019), Yulianto et al. (2019), Hong et al. (2018), Huang et al. (2023) Pastrana and Suarez-Tangil (2019), Feng et al. (2022), Tahir et al. (2019), Romano et al. (2020), Rauchberger et al. (2018), Kharraz et al. (2019), Musch et al. (2019), Varlioglu et al. (2020), Hong et al. (2018), Bijmans et al. (2019), Zhang et al. (2023)	Meland et al. (2019), Lachtar et al. (2020), Feng et al. (2020), Saad et al. (2018), Bian et al. (2019), Bian et al. (2020), Tekiner et al. (2021)	Gomes and Correia (2020), Tanana and Tanana (2020), Naseem et al. (2021), Gomes et al. (2020), Zimba et al. (2019), Carlin et al. (2020), Shih et al. (2020), Aktepe et al. (2020), Hernandez-Suarez et al. (2022), Wang et al. (2021), Mansor et al. (2020), Rodriguez et al. (2018), Carlin et al. (2018), Mani et al. (2020), Tommasi et al. (2022), Dinulica and Cosma (2020), Zimba et al. (2020), Abbasi et al. (2023), Romano et al. (2020), Belkin et al. (2019) Mani et al. (2023) Li et al. (2022), Rajba and Mazurczyk (2022), Chow et al. (2022), Zhang et al. (2021), Bhansali et al. (2022)	Caviglione et al. (2021), Varlioglu et al. (2022), Belkin et al. (2019), TGlobal Initiative Against Transnational Organized Crime (2022) Sriman et al. (2023)
Mobile	Cybercriminals target mobile devices to mine cryptocurrencies.	Global Initiative Against Transnational Organized Crime (2022), Dashevskyi et al. (2020)	Tekiner et al. (2021)		Gera et al. (2021), New fileless crypto-miner targets corporate networks across the world (2018)
IoT	Cybercriminals compromised IoT devices to use their computational power to mine cryptocurrencies.	RGlobal Initiative Against Transnational Organized Crime (2022), Carrillo-Mondejar et al. (2020), Haseeb et al. (2020), Pour et al. (2019)	Celdrn et al. (2023), Lee et al. (2022), Tekiner et al. (2021)	Gomes and Correia (2020), Swedan et al. (2018), Mansor et al. (2020), Ngo et al. (2021), Zareh and Shahriari (2018), Breitenbacher et al. (2019), Ahmad et al. (2019)	Caviglione et al. (2021), Cybersecurity industry market report (2020), Borys et al. (2022), Khang et al. (2021), Pan et al. (2021), New fileless crypto-miner targets corporate networks across the world (2018), Li and Wu (2018), Torbet (2019), Cybersecurity Industry (2018)
File	Malware sent (via e-mail for example) gains access to user’s file and system computer resources (i.e. memory) Belkin et al. (2019).	Pastrana and Suarez-Tangil (2019), Xiao et al. (2023)	Celdrn et al. (2023) Franco et al. (2023), Pott et al. (2023), Tekiner et al. (2021)	Aljehani and Alsuwat (2022), Tanana and Tanana (2020), Gomes et al. (2020), Zimba et al. (2020), Yazdinejad et al. (2020), Ning et al. (2019), Berecz and Czibula (2019) Sharma and Swapna (2023) Li et al. (2022)	Cybersecurity industry market report (2020), Global Initiative Against Transnational Organized Crime (2022), Khang et al. (2021), Munish (2020)
Cloud	Attackers search through the code or files of an organization in the hope of finding the application programming interface (API) keys to access the cloud service. Following this step, they can use CPU resources to mine cryptocurrency, leading to massive increases in electricity and computer power Belkin et al. (2019)	Li et al. (2022)	Fargo et al. (2020), Zhang et al. (2023), Tekiner et al. (2021)	Tanana and Tanana (2020)	Global Initiative Against Transnational Organized Crime (2022), Jayasinghe and Poravi (2020)
Other	Examples of such include cyber-physical systems.	Mani et al. (2018)	Zimba et al. (2019)	Zheng et al. (2022), Karn et al. (2021), Sergeev et al. (2019), Fargo et al. (2020), Pastor et al. (2020), Ahmad et al. (2019), Tanana (2020), Vesely and Zadnik (2019)	New fileless crypto-miner targets corporate networks across the world (2018), Cybersecurity industry market report (2020), Capuano et al. (2022)

In addition to the economic consequences of cryptojacking, the impact of cryptomining malware on users’ devices are also noted. In 2017, over 500 million computers were estimated to be affected by cryptomining malware all over the world, and 220 of the top 100,000 most visited websites included a background cryptomining script (Aktepe et al., 2020). In 2018, studies indicate that cryptomining malware affected 42% of organizations around the world (Cybersecurity Industry, 2018), including high profile companies such as Starbucks, YouTube, Los Angeles Times and Tesla (via poor AWS configuration) and other online video sites (Carlin et al., 2020). In line with increasing economic value of cryptocurrencies, the literature suggests a sharp rise in cryptojacking the following year. In 2019, 850,000 unique infections of Retadup – a cryptomining malware botnet – were found and neutralised through a raid on the command-and-control (C&C) server (Torbet, 2019). Finally, in 2020 cryptomining malware impacted 11% of the organizations worldwide, with XMRig been the most prominent cryptomining malware impacting 7% organizations worldwide (Cybersecurity industry market report, 2020).

Although papers focus on a range of figures to measure the economic impact cryptojacking, common themes can be determined. The most common economic consequences as found in the literature were degradation of computer performance and potential reduction of hardware lifetime (Khang et al., 2021; Gera et al., 2021; Global Initiative Against Transnational Organized Crime, 2022; Gomes et al., 2020; Gomes and Correia, 2020; Saad et al., 2019; Pastrana & Suarez-Tangil, 2019; Sergeev et al., 2019; Shih et al., 2020; Belkin et al., 2019; Sriman et al., 2023; Mani et al., 2023; Li et al., 2022); high CPU usage and power consumption (Khang et al., 2021; Gera et al., 2021; Allen et al., 2022; Gomes et al., 2020; Gomes and Correia, 2020; Saad et al., 2019; Shih et al., 2020; Hong et al., 2018; Carlin et al., 2020; Sriman et al., 2023), and insecure data (Sriman et al., 2023). Additionally, a number of secondary effects of cryptojacking were reported in the literature, such as: increased energy bills (Global Initiative Against Transnational Organized Crime, 2022; Pastrana & Suarez-Tangil, 2019; Belkin et al., 2019; Sriman et al., 2023), increased ecological footprint and environmental impact (Pastrana & Suarez-Tangil, 2019), and poor web experience for end-users (Belkin et al., 2019). These findings, however, were speculatively derived; as such it is important to consider their provisional nature and to conduct further investigation for their validation.

As has been alluded to, cryptomining attacks target a range of victims. From the extracted studies, three main victim groups can be identified: individual end-users; enterprises; and other entities, which include governmental and non-commercial organisations. Table 4 details the victim groups focused on by individual papers in the corpus. As illustrated in the table, studies on cryptojacking are concentrated around end-users, yet cryptomining malware both target and impact a wide range of victims, including enterprises and even critical infrastructure systems. This demonstrates the broad scope and potential impact of such attacks, as threat actors exploit both personal and organizational computing resources to mine cryptocurrency illicitly. The inclusion of critical infrastructure among the targets is particularly alarming, highlighting the growing sophistication and ambition of these cyber threats.

**Table 4:** Targets of cryptomining malware attacks

Target	Papers	
End-user	The device attacked belongs to an individual	Aljehani and Alsuwat (2022), Karn et al. (2021), Wang et al. (2021), Mansor et al. (2020), Rodriguez et al. (2018), Carlin et al. (2018), Tommasi et al. (2022), Allen et al. (2022), Carrillo-Mondejar et al. (2020), Zheng et al. (2022), Hernandez-Suarez et al. (2022), Yazdinejad et al. (2020), Dinulica and Cosma (2020), Ning et al. (2019), Sergeev et al. (2019), Burgess et al. (2019), Saad et al. (2019), Lachtar et al. (2020), Zimba et al. (2020), Caviglione et al. (2021), Meland et al. (2019), Khang et al. (2021), New fileless crypto-miner targets corporate networks across the world (2018), Claire (2019), Belkin et al. (2019), Torbet (2019), Borys et al. (2022), Konoth et al. (2018), Bijmans et al. (2019), Shih et al. (2020), Pastrana and Suarez-Tangil (2019), Belkin et al. (2019), Gera et al. (2021), Li and Wu (2018), Haseeb et al. (2020), Caprolu et al. (2021), Gomes et al. (2020), Gomes and Correia (2020), Tanana (2020), Tanana and Tanana (2020), Zimba et al. (2019), Zimba et al. (2019), New fileless crypto-miner targets corporate networks across the world (2018), Europol (2019), Wilczek (2019), Study highlights rise in illegal cryptomining malware (2018), Sharma and Swapna (2023), Wrieden and Vassilakis (2023), Mani et al. (2023), Goyal and Matta (2023), Li et al. (2022), Celdrn et al. (2023), Hu et al. (2023), Abbasi et al. (2023), Bill Toulas (2023), Guangquan et al. (2023) Feng et al. (2022), Rajba and Mazurczyk (2022), Bhansali et al. (2022), Kharraz et al. (2019), Musch et al. (2019), Zhang et al. (2021), Rauchberger et al. (2018), Chow et al. (2022), Dashevskyi et al. (2020), Jayasinghe and Poravi (2020), Lee et al. (2022), Pott et al. (2023), Bian et al. (2020), Bian et al. (2019), Hong et al. (2018), Bijmans et al. (2019), Chow et al. (2022), Zhang et al. (2023)
Enterprise	The target of the attack are commercial organisations	Gomes et al. (2020), Pastrana and Suarez-Tangil (2019), Borys et al. (2022), Ahmad et al. (2019), Swedan et al. (2018), Vesely and Zadnik (2019), New fileless crypto-miner targets corporate networks across the world (2018), Korolov (2018), Study highlights rise in illegal cryptomining malware (2018), Li et al. (2022), Franco et al. (2023), Huang et al. (2023), Jayasinghe and Poravi (2020), Zhang et al. (2023)
Other	Attack targets are critical organisations such as state/government agencies and service providers	Claire (2019), Fargo et al. (2020)

**Table 5:** Summary of cryptojacking detection methods identified

Type of detection	Description	Papers
(Supervised) Machine Learning	e.g. Decision Trees, Random Forests, Gradient Boosting, K Nearest Neighbor, SVM, Logistic Regression	Karn et al. (2021), Ngo et al. (2021), Wang et al. (2021), Tanana (2020), Borys et al. (2022), Swedan et al. (2018), Mansor et al. (2020), Vesely and Zadnik (2019), Rodriguez et al. (2018), Carlin et al. (2018), Tanana and Tanana (2020), Mani et al. (2020), Tommasi et al. (2022), Allen et al. (2022), Gomes and Correia (2020), Carrillo-Mondejar et al. (2020), Berecz and Czibula (2019), Caprolu et al. (2021), Sharma and Swapna (2023), Li et al. (2022), Abbasi et al. (2023), Kharraz et al. (2019), Dashevskyi et al. (2020), Tahir et al. (2019), Saad et al. (2018), Zhang et al. (2023), Pott et al. (2023), Xiao et al. (2023)
Deep Neural Networks	e.g. Convolutional Neural Network (CNN), Recurrent Neural Networks (RNN), Long Short Term Memory Network (LSTM), Graph Neural Network, Autoencoder, Variational Autoencoder	Aljehani and Alsuwat (2022), Zheng et al. (2022), Karn et al. (2021), Naseem et al. (2021), Hernandez-Suarez et al. (2022), Borys et al. (2022), Yazdinejad et al. (2020), Dinulica and Cosma (2020), Ning et al. (2019), Mani et al. (2020), Sergeev et al. (2019), Sanda et al. (2024), Li et al. (2022), Huang et al. (2023), Mani et al. (2023), Feng et al. (2020), Feng et al. (2022), Pour et al. (2019), Bhansali et al. (2022), Chow et al. (2022)
Heuristic	e.g. Matching URLs present in code against blacklist	Zareh and Shahriari (2018), Breitenbacher et al. (2019), Burgess et al. (2019), Saad et al. (2019), Lachtar et al. (2020), Yulianto et al. (2019), Zheng et al. (2022), Aktepe et al. (2020), Fargo et al. (2020), Franco et al. (2023), Bian et al. (2020)
Other	Clustering	Gomes et al. (2020)
	Dendritic Cell Algorithm	Ahmad et al. (2019)
	Statistical analysis (K-S test)	Hu et al. (2023)

**Fig. 4:**
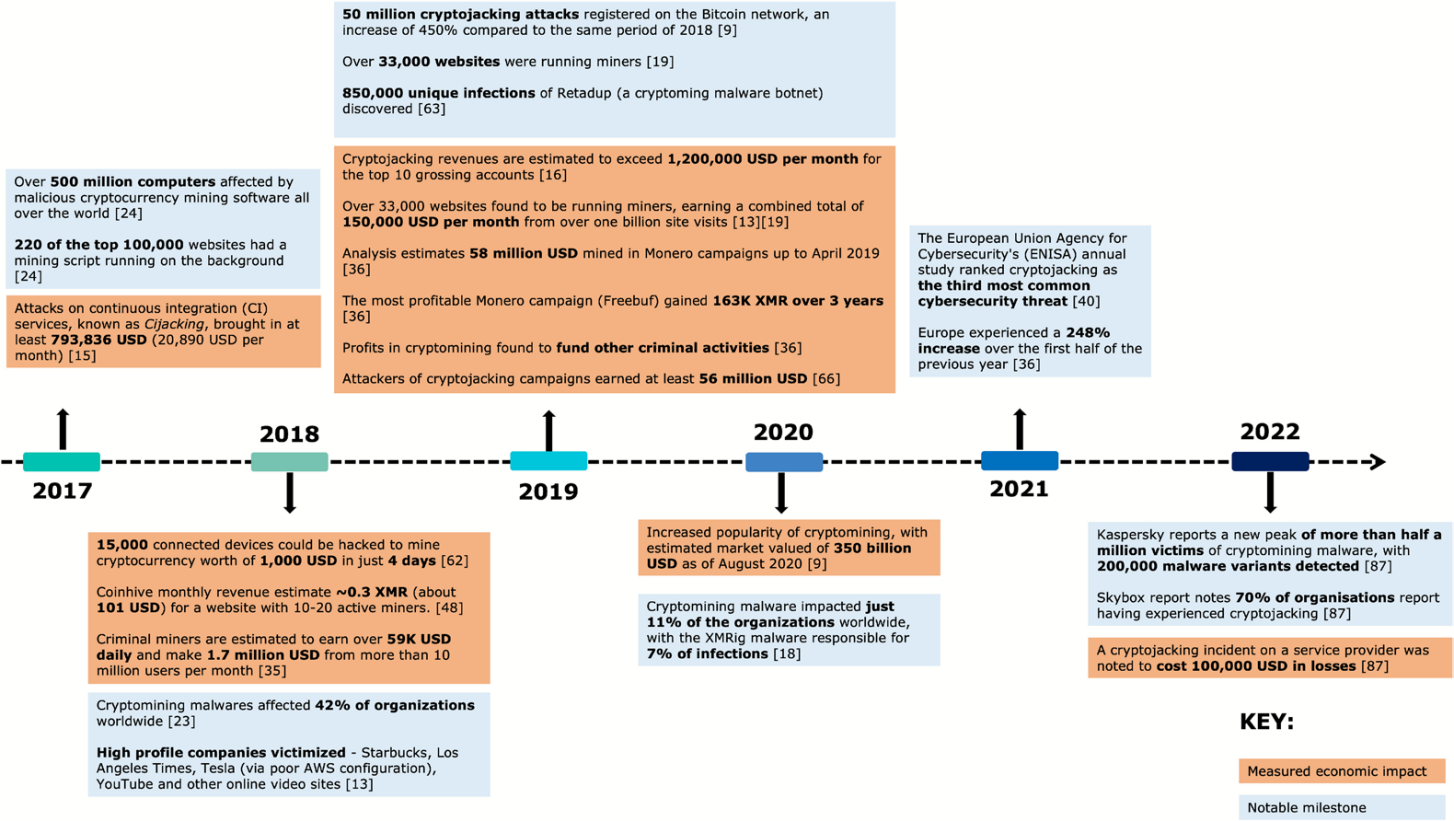
Timeline of the overall (economic and measured) impact of cryptomining malware

As many of the reviewed papers focus on cryptojacking detection – in line with our third research question – we now focus on cryptomining malware detection approaches explored in the literature ).

### Malware detection

Of the 119 articles included in the final analysis, half (67) focused on detecting cryptomining malware using a range of methods. Detection techniques explored in the literature, as well as the features used in these approaches, are collated in Tables 5 and 6. These methods adopted are largely dependent on the chosen features utilised by the model, and are grouped into *static*, *dynamic*, *network*, and *hybrid* approaches. Static approaches do not involve the running of the executable file, relying instead on features relating to code structure and other metadata. In contrast, dynamic approaches involves the actual execution of the malware script within a controlled sandbox environment. In doing so, the behaviour of the script – including memory usage and network activity – can be observed. The latter is of particular note in a number of papers, which focus on detecting cryptomining malware in corporate networks, with successes in utilising network-based features such as TCP/IP packet traffic data as detection features (Hu et al., 2023); a particularly advantageous approach given its centralised, user data-agnostic nature. Utilising these features, approaches have ranged from leveraging heuristics for detection (Hong et al., 2018; Dinulica & Cosma, 2020; Aktepe et al., 2020) to implementing machine learning classification models; such as Support Vector Machine (SVM) (Sharma & Swapna, 2023); Random Forest (RF) (Caprolu et al., 2021); and convolutional neural network (CNN)-based approaches (Pour et al., 2019); amongst others (Table 5 ).

**Table 6:** Summary of features used in cryptojacking detection

Types of features	Description	Papers
Dynamic	e.g. Runtime analysis, JavaScript APIs called, CPU Usage Metrics	Karn et al. (2021), Naseem et al. (2021), Hernandez-Suarez et al. (2022), Zareh and Shahriari (2018), Tanana and Tanana (2020), Saad et al. (2019), Zimba et al. (2020), Dinulica and Cosma (2020), Ning et al. (2019), Zimba et al. (2019), Rodriguez et al. (2018), Hong et al. (2018), Carlin et al. (2018), Tanana (2020), Mani et al. (2020), Fargo et al. (2020), Guangquan et al. (2023), Pott et al. (2023), Hong et al. (2018), Varlioglu et al. (2020), Tahir et al. (2019), Bian et al. (2019), Bian et al. (2020)
Static	e.g. Code analysis, Abstract Syntax Tree	Zheng et al. (2022), Wang et al. (2021), Breitenbacher et al. (2019), Burgess et al. (2019), Saad et al. (2019), Zimba et al. (2020), Ahmad et al. (2019), Yazdinejad et al. (2020), Swedan et al. (2018), Lachtar et al. (2020), Romano et al. (2020), Mani et al. (2020), Yulianto et al. (2019), Zheng et al. (2022), Aktepe et al. (2020), Kharraz et al. (2019), Pour et al. (2019), Romano et al. (2020)
Hybrid	Combination of Static, Dynamic and/or Network	Aljehani and Alsuwat (2022, 2022), Ngo et al. (2021), Tommasi et al. (2022), Gomes et al. (2020), Carrillo-Mondejar et al. (2020), Berecz and Czibula (2019), Caprolu et al. (2021), Sharma and Swapna (2023), Franco et al. (2023), Celdrn et al. (2023), Li et al. (2022), Sanda et al. (2024), Abbasi et al. (2023), Saad et al. (2018), Dashevskyi et al. (2020), Musch et al. (2019)
Network	e.g. Domains visited, Size and frequency of TCP packets	Borys et al. (2022), Vesely and Zadnik (2019), Allen et al. (2022), Franco et al. (2023), Huang et al. (2023), Hu et al. (2023), Feng et al. (2020), Feng et al. (2022), Lee et al. (2022), Chow et al. (2022), Zhang et al. (2023), Rauchberger et al. (2018), Zhang et al. (2021), Bijmans et al. (2019)

### Security recommendations

Most articles captured through this review (88) included recommendations for better security practices and mitigation strategies to curb cryptojacking. These were broadly categorised according to their associated stakeholder, namely: end-users; enterprises; security researchers; and governmental organisations. A brief description of each category is included below in Table 7, alongside the most common suggestions. Recommendations also covered a wide range of competencies, including technical implementation, legal solutions and cybersecurity education.

The majority of articles considered and proposed technical approaches - in particular, the development of software tools to detect and inhibit cryptojacking. These took a variety of approaches, as have been mentioned in the previous section. The adoption of more targeted policies, both by governments and organisations, also featured in the literature. For enterprises, the deployment of tighter operational security (OPSEC) policies was suggested, particularly where IoT device use was concerned (Khang et al., 2021). At the policy level, governments were encouraged to engage in international co-operation through multilateral agreements to deal with cybercrime in general, including cryptojacking ( T Global Initiative Against Transnational Organized Crime , 2022). Acknowledging the inherent asymmetry between attackers and defence in cyberspace, consideration is given to the possible evolutiionary pathways of the threat landscape.

**Table 7:** Summary of security recommendations, categorised by stakeholder

Stakeholder	Recommendations		Reference
End-users (Lay users of computer devices; employees)	Increased consumer cybersecurity awareness		Occasional Paper - Closing the Crypto Gap - Guidance for Countering North Korean (2019), Borys et al. (2022), Vuuren et al. (2019)
	Usage of Tor network		Mani et al. (2018)
Enterprises (Business organisations, ranging from SMEs to critical national infrastructure (CNI))	Incorporate defence-in-depth and honeypots within network design		New fileless crypto-miner targets corporate networks across the world (2018), Tok et al. (2022), Khang et al. (2021)
	Corporate employee training		New fileless crypto-miner targets corporate networks across the world (2018), Khang et al. (2021), Vuuren et al. (2019)
	Use of sub-resource integrity and content security policy by website admins		Burgess et al. (2019), Carlin et al. (2020)
	Improved OPSEC policies		New fileless crypto-miner targets corporate networks across the world (2018), Khang et al. (2021)
	Adoption of anti-virus/malware software		Meland et al. (2019)
	Analysis of mining traffic via network deep packet inspection		Zheng et al. (2021)
	Wide-spread adoption of consent-based web mining		Hong et al. (2018)
	Auditing of process creations and command line activities		Varlioglu et al. (2022)
	More focus on IoT security		Borys et al. (2022)
Security researchers (academics and analysts from cybersecurity firms)	Improving detection techniques using:	Machine learning techniques	Borys et al. (2022), Konoth et al. (2018), Aljehani and Alsuwat (2022), Gomes and Correia (2020), Tanana and Tanana (2020), Gomes et al. (2020), Burgess et al. (2019), Swedan et al. (2018), Hernandez-Suarez et al. (2022), Pastrana and Suarez-Tangil (2019), Saad et al. (2019), Wang et al. (2021), Dinulica and Cosma (2020), Zheng et al. (2021), Carrillo-Mondejar et al. (2020), Ngo et al. (2021), Zareh and Shahriari (2018), Breitenbacher et al. (2019), Yazdinejad et al. (2020), Berecz and Czibula (2019), Zheng et al. (2022), Karn et al. (2021), Vesely and Zadnik (2019), Caprolu et al. (2021)
		Side-channel analysis	Tanana and Tanana (2020), Hong et al. (2018), Sergeev et al. (2019), Korolov (2018)
		Explainable AI methods	Caviglione et al. (2021), Karn et al. (2021)
		Cryptographic function analysis	Sergeev et al. (2019)
	Development of browser extensions to detect cryptojacking scripts		Naseem et al. (2021), Carlin et al. (2020), Aktepe et al. (2020), Meland et al. (2019), Yulianto et al. (2019), Dinulica and Cosma (2020), Romano et al. (2020), Belkin et al. (2019)
	Novel cryptojacking malware classification systems		Mansor et al. (2020), Mani et al. (2020), Carrillo-Mondejar et al. (2020), Ahmad et al. (2019)
	Preparation of trusted datasets for security research		Caviglione et al. (2021)
	Improved threat modelling		Khang et al. (2021)
	Leverage social media such as Twitter for threat intelligence		Varlioglu et al. (2022)
	Greater focus on IoT security		Borys et al. (2022)
Governmental organisations (public sector and state agencies)	Improved cybercrime strategy including co-operation between law enforcement and service providers		Europol (2019), Torbet (2019), Vuuren et al. (2019)
	Regulation of cryptocurrency usage, generation and exchange		Eskandari et al. (2018), Claire (2019)
	Greater focus on cyber diplomacy and effective international cooperation		Munish (2020), Vuuren et al. (2019)

### Future trends

This section presents potential future trends as reported in the literature. Overall, the literature seems to agree that cryptomining malware will remain a large and imminent threat in the future. Both illicit and legal mining activities may be more common as the value of cryptocurrency continues to rise. There is also a consensus of a predicted increase in the spread of cryptomining malware in various ways (not just via JavaScript), but the literature predicts a move away from file-based cryptojacking. In terms of browser-based cryptojacking, despite the fact that CoinHive was shut down in March 2019, cybercriminals are simply switching towards different services such as CryptoLoot and JSECoin. Some sources also predict malicious website owners may combine cryptojacking with online advertisement to increase their overall revenue from websites (Saad et al., 2018). This also points towards the usage of more organised services offered both for cryptojacking itself i.e. cybercriminals increasingly organise their cryptomining campaigns (Bijmans et al., 2019) as well as in terms of distribution. This may be noticeable by offenders using using third-party app installations (Bill Toulas, 2023) or even using Botnet as a Service (Khang et al., 2021). On the topic of botnets, a paper predicted a potential evolution of how botnet traffic might be optimised by hiding the communication in the Bitcoin protocol which could be used for cryptojacking (Pan et al., 2021).

The literature suggests that the level of sophistication of cryptomining malware may increase and a rapid proliferation of new cryptojacking variants with minimal investment in infrastructure; however, there have been many hypotheses as to how exactly perpetrators may achieve that. Some examples of this include predicted rise in cryptojacking injections, adopting worm-like properties (Europol, 2019) moving from using CPU resources to abusing GPU resources (Tanana & Tanana, 2020) (Li et al., 2022) and optimisations of browser mining (Hong et al., 2018). The increased sophistication also extends to the obfuscation techniques, cryptomining malware may be using - this extends from using AI within the malware to evade detection in various ways - from code obfuscation to using automated scripts to change the mining pool IP more often (hourly) (Hu et al., 2023). A paper studying real world data based on our hierarchy of evidence, also identified the 3 most common obfuscation strategies cryptominers use: limiting CPU use, hiding code and hiding payload (Hong et al., 2018).

In terms of targets of cryptojacking, the research suggests cyber-physical systems, IoT devices, mobile devices and the cloud may be increasingly more targeted in the future (Table 8). One article even suggests mining on devices performing crowdsensing such as via ElectroSense network, meaning the cryptomining malware would leverage the collective devices of a decentralised network (Celdrn et al., 2023). Further to the mentioned vulnerable technologies, one highly speculative paper proposed a threat where cryptojacking could lead to the monetisation of human consciousness, drawing a parallel with current wearable health technology reward programs (Sempreboni & Viganò, 2018). Moreover, the literature predicts the shift from user-based targets to more corporate targets due to higher rewards. This may mean that malicious insiders could become the perpetrators (Caprolu et al., 2021).

Monero is predicted to continue being the most profitable cryptocurrency for malicious cryptomining operations, largely due to its privacy-centric design and CPU-minable algorithm. In contrast with the SHA-256 algorithm used for Bitcoin mining, which requires specialised application-specific integrated circuit (ASIC) hardware, Monero utilises the RandomX proof-of-work algorithm, which is optimised for general-purpose CPUs (Kumar et al., 2017). RandomX’s compatibility with both intensive and mobile computing has expanded the attack surface for cryptojacking across a wide range of devices; including desktops, servers, and increasingly, mobile phones. Moreover, Monero’s built-in privacy features, such as ring signatures and stealth addresses, make it especially appealing for illicit use, as they obscure transaction traces on the blockchain, effectively shielding both the recipients and the amounts involved (Kumar et al., 2017; Zhang, 2023). Hence, combined with the widespread proliferation of the Internet of Things (IoT), Monero offers a higher return on yield comparative to other cryptocurrencies. This may likely solidify its dominance as the cryptocurrency of choice for cryptojacking being employed in numerous malware families, including XMRig-based variants – frequently adapted to exploit both individual devices and large-scale botnets (Pastrana & Suarez-Tangil, 2019; Alexander Reed, 2025).

From the perspective of perpetrators, grey literature reports that cryptojacking continues to become a global issue - in particular, they foresee a spread of such activities in the developing world, such as in the African region. This may impact the spread of hubs of organised, cyber-enabled criminal networks and present a novel means of financing, (Global Initiative Against Transnational Organized Crime 2022; Vuuren et al., 2019).

In addition to security recommendations outlined in the section above, grey literature especially calls for future regulation and adoption on the global scale to help shape and mitigate the threat cryptomining malware presents (Claire, 2019). Particularly, the literature points out that the cryptocurrency regulatory environment is fragmented and as such the absence of a unified international regulatory framework may give rise to cryptocurrency safe havens, wherein terrorist and transnational criminal organisations are able to operate with relative impunity in jurisdictions characterised by minimal oversight. Indeed, malicious actors are already able to hold and transfer cryptocurrency openly, provided their activities are conducted through exchanges situated beyond the reach of more rigorously regulated states (Thomas Ott, 2018).

**Table 8:** Summary of future trends as reported in the literature

Trend group	Trend	Description	Papers
Overall	Increased prevalence of cryptomining malware	The prevalence of cryptomining malware may increase due to many factors including - the anonymity of perpetrators and low barriers of entry, cryptocurrency remaining the main method of currency on the dark web, and the development of vectors for spreading the malware with minimal investment in infrastructure	Europol (2019), Aktepe et al. (2020), Tanana (2020), Varlioglu et al. (2022), Carrillo-Mondejar et al. (2020), Planbureau (2019), Lachtar et al. (2020), Khang et al. (2021), Claire (2019), Caprolu et al. (2021), Aktepe et al. (2020), Pan et al. (2021), Hong et al. (2018), Zimba et al. (2019), Zimba et al. (2020), Gomes et al. (2020), Sharma and Swapna (2023), Sriman et al. (2023), Saad et al. (2018)
Comparison to other cybercrime operations	Ransomware	Ransomware and cryptomining malware are currently two large streams of funding for cybercriminals. Malicious cryptominers may overtake ransomware as money generators or even combine the two - carry out a ransomware attack post cryptomining	Europol (2019), Varlioglu et al. (2022), Gera et al. (2021)
Vulnerable technologies	Cyber-physical systems (CPS)	As the infrastructure is moving towards smart technology, the rise of CPS is imminent, making them a target	Caviglione et al. (2021), Ahmad et al. (2019)
	Internet of Things (IoT)	Increase in IoT devices likely to lead to more IoT-focused cryptomining malware for even low-end IoT devices	Ngo et al. (2021), Zheng et al. (2021), Breitenbacher et al. (2019), Swedan et al. (2018), Mansor et al. (2020), Haseeb et al. (2020), Carrillo-Mondejar et al. (2020), Ahmad et al. (2019), Zheng et al. (2021), Caviglione et al. (2021), Celdrn et al. (2023)
	Mobile devices	Increase of mobile malware targeting mobile devices as the number of devices grows every year	Europol (2019) Caviglione et al. (2021) Sharma and Swapna (2023)
	Cloud computing	Further increase in cloud-based computing may result in more cryptomining malware targeting cloud systems. This may also lead to increased virtualisation techniques and thus more VM malware	Berecz and Czibula (2019), Fargo et al. (2020), Sergeev et al. (2019)
New targets	Increased corporate targets	The move from targeting end users to corporate targets including DevOps platforms may present higher monetary reward to cybercriminals	New fileless crypto-miner targets corporate networks across the world (2018), Li et al. (2022), Caprolu et al. (2021)
Obfuscation	Increased sophistication	Already, antivirus programs have difficulties detecting cryptomining malware,this is likely to increase as obfuscation methods criminals use are likely to become more sophisticated. Some of the common methods may include the use of iframe elements in web pages and the use of pre-existing, sophisticated cryptomining malware services	Zheng et al. (2022), Tommasi et al. (2022), Pastrana and Suarez-Tangil (2019), Gomes et al. (2020), Goyal and Matta (2023), Hu et al. (2023), Bian et al. (2020), Bian et al. (2019), Hong et al. (2018)

## Discussion

This paper presents a comprehensive discussion on cryptomining malware, analyzing historical trends, evaluating current detection techniques and security recommendations, and exploring potential future developments in this evolving cybersecurity threat. The literature shows that for the lay end-user, the primary impact of illegal mining is a noticeable decline in device performance and a shortened hardware lifespan, making cryptojacking a high-impact, victim extending form of cybercrime. During testing, CPU temperatures exceeded the safe threshold of 82 °C, posing a risk to the device’s stability and longevity (Franco et al., 2023). Additionally – given the substantial energy consumption of the cryptomining computational process – cryptojacking enables criminals to pass these costs onto individuals via their energy bills, increasing their profit margins compared to conventional mining, and compounding the burden on victims (New fileless crypto-miner targets corporate networks across the world, 2018). Given increasingly constrained availability – due to geopolitical tensions and supply chain disruption – of critical materials necessary for computing components such as lithium, cobalt and rare earth elements, the collective impact of premature wear of devices caused by cryptomining malware may exacerbate already strained demand for these resources (Althaf & Babbitt, 2021). Similar concerns are mirrored in from an environmental impact and digital sustainability standpoint – although such concerns have been ascribed to the cryptocurrency ecosystem at large (Pastrana & Suarez-Tangil, 2019). Additionally, in regions where energy is scarce or expensive, or when infrastructure is particularly vulnerable, the cumulative impact of cryptojacking could be substantial. Finally, some malicious malware do not only uses the system to mine the cryptocurrency, but also to access the financial data without the user’s knowledge, making the data insecure (Sriman et al., 2023).

For corporate targets, there is the additional issue of increased latency on local networks, where multiple infected computers may be transmitting mining traffic. This network effect, along with the decrease in computational power and the rise in costs, poses a considerable threat to business processes and continuity, particularly in low-latency environments such as the financial sector with lower tolerance to such disruption. This can have a significant effect on profits, highlighting the great need for mitigation strategies (New fileless crypto-miner targets corporate networks across the world, 2018). With the rise of corporate Bring-Your-Own-Device (BOYD) schemes, the obfuscation attempts detailed in the literature highlight the criticality for detection mechanisms to anticipate and neutralise cryptojacking attempts.

Educational institutions also find themselves in a vulnerable position, due to the potential of insider threats in the forms of students taking advantage of their IT systems to perform cryptomining, particularly with some form of legal protection (Korolov, 2018) This, combined with the difficulty in detection, leads to the generally held view that most instance of cryptomining malware goes unreported (Blythe & Johnson, 2021). This is consistent with the wider cybercrime ecosystem; a fact which points to a potential underestimation of the true prevalence of cryptomining malware within such systems. Furthermore, despite attempts by various researchers, there does not appear to be a systematic and agreed methodology for evaluating the economic impact of cryptojacking. However, we found that there is a correlation between cryptojacking activity and cryptocurrency market fluctuations (See Fig. 2). For instance, the decline in cryptojacking incidents following the 2019 shutdown of Coinhive, a major browser-based mining service, coincided with a broader downturn in crypto markets. Conversely, the 2021–2022 surge in cryptojacking interest aligned with a surge of cryptocurrency valuations. This pattern might suggest that cryptojacking is a financially opportunistic behaviour, responsive to market incentives. An emerging and under-explored dimension of the cryptojacking threat landscape is the role of artificial intelligence (AI) in expanding access to high-performance computing resources, particularly GPUs, which have become increasingly attractive targets for cryptojacking attacks (Xiao et al., 2023). At the same time, the rapid deployment and adoption of AI – in particular, commercially available large language models (LLMs) – malware detection techniques have necessarily increased in sophistication and robustness over the years. While initial approaches largely relied on heuristic features – such as reliance on publicly available blacklists containing URLs of known cryptomining pools (such as NoCoin and MinerBlock) (Hong et al., 2018; Zareh & Shahriari, 2018; Eskandari et al., 2018; Dinulica & Cosma, 2020); or commonly used cryptomining scripts and WebAssembly modules on browser-based sessions (Aktepe et al., 2020) – such methods are not robust to the evolving threat landscape: they are largely dependent on community information-sharing; and are fragile against the various obfuscation mechanisms utilised by attackers (Wang et al., 2021). Building on these, work has begun to look at runtime behavioural features: employing dynamic features such as CPU usage (Konoth et al., 2018) and block header analysis (Zareh & Shahriari, 2018); with some opting to consider hybrid features for greater sophistication (Zimba et al., 2019; Dinulica & Cosma, 2020). In line with advances in machine learning (ML) models – particularly those featuring deep neural networks (DNNs) – cryptojacking detection solutions have seen rapid advancement, being deployed for binary and even multi-class classification approaches; evidenced by their high representation in Table 5. In taking into account a wider scope of hardware and software performance indicators to develop highly dimensional classification systems, such models offer an element of robustness to the intrinsic challenge of detection in light of obfuscation attempts, generally achieving high precision and recall rates of greater than 90.

Users are beginning to see the impact of better and more accurate detection mechanisms, being better able to identify and subsequently avoid malicious websites and software. Therefore, as cryptomining malware continues to evolve in both form and function, malware detection remains a key area of research. The detection mechanisms and security recommendations identified should also be continuously revised as with any malware, cryptojacking malware is an arms race between security practitioners and the bad actors who continue to increase the stealthiness of the malware and its sophistication of detection evasion techniques.

Discussion within the literature reflects the multidimensional approach required for dealing with the phenomenon of cryptojacking. While technical perspectives, in particular detection, have generated ample conversation, there is less of a focus on the responsibilities of governments for closing potential legal loopholes.

A key mitigation that was not widely discussed by papers is that majority of the larger browsers such as Chrome, Firefox, and Safari, offer built-in protections against cryptomining malware. This is a massive step towards end-user safety as browser-based cryptomining malware has been found to be one of the wider spread malware. However, browser-based mining represents only one manifestation of cryptomining malware; notably, our investigation into emerging trends indicates a likely shift in cryptojacking targets towards mobile devices, cyber-physical systems, cloud infrastructures, and IoT environments. Furthermore, although some widely used browsers incorporate built-in protective features, this functionality is not universally available across all platforms. Existing literature emphasises that such browser-level safeguards provide only a baseline defence rather than comprehensive protection. Enhanced mitigation strategies typically involve supplementary measures such as browser extensions, plug-ins, and ad-blockers. However, empirical evidence underscores the limitations of these approaches: a recent study assessing countermeasures within Google Chrome based on real-world data revealed poor detection efficacy, failing to identify cryptojacking scripts in over 50% of cases (Rajba & Mazurczyk, 2022). Furthermore, when miners were subjected to minor modifications, blocking solutions exhibited an even higher failure rate, exceeding 60%.

At least one work notes that US law currently criminalises cryptojacking only once a threshold of 5,000 USD in illegal profits over a one-year period on a single computer (Carlin et al., 2020), a scenario which does not take into account the distributed nature of cryptocurrency mining - cybercriminals may well surpass this amount without meeting this threshold on a single device. This distributed model allows cybercriminals to evade prosecution under current thresholds, despite generating substantial illicit profit overall. The geographic distribution of cryptojacking appears to follow other more established forms of cybercrime (Allen et al., 2022): as such, suggestions to improve international legal cooperation on cryptojacking are subsumed into the broader conversation on international cybercrime, such as within the recent United Nations Convention against Cybercrime (United nations convention against cybercrime, 2024).

The articles reported in Table 8 predicted the increased prevalence of cryptojacking in the future due to the low barrier of entry, low detection rates of cryptomining malware in the wild, and high monetary rewards. It is also worth re-emphasising that the cryptocurrency market is a highly volatile one and as such future trends will reflect that. Most of the work that was assessed in this research happened during the peak of cryptocurrency; however, since the cryptocurrency crash in 2021/2022 where it has been estimated that the cryptocurrency industry decreased by two trillion US dollars (Kalhoro et al., 2021), these estimates may change.

The literature also predicts the widespread of cryptojacking in emerging technologies such as Internet of Things (IoT), cyber-physical systems (CPS), and cloud technologies. As the world becomes more interconnected, the volume of crime opportunities will increase. It is vital to develop suitable detection and prevention measurements, especially in the fields such as IoT, where cyber-hygiene is particularly lacking (Oravec, 2017). In response to this, the literature encourages the community to focus more of its efforts into researching the threats and developing protection mechanisms for file-based cryptojacking, particularly those strains that target mobile devices, CPS and IoT. Lastly, there are suggestions within the literature of future attacks emerging of a more targeted (i.e. more sophisticated) nature, in particular targeting corporate resources. This – combined with predictions of increasingly sophisticated obfuscation techniques for network and device activity – suggests a dire outlook for development and implementation of detection and mitigation procedures for illegal cryptomining.

The targeting of wide range of victims from end-users, corporate spaces, to cyberphysical systems, IoT devices, and even critical national infrastructure by cryptomining malware elevates this phenomenon from a financially motivated cybercrime to a high-impact threat with systemic consequences. From a crime science perspective, such attacks exemplify the convergence of opportunistic theory and deterrence theory (or lack of thereof), where actors exploit low-risk, high-reward opportunities within poorly regulated digital environments (Cornish & Clarke, 2014; Pratt et al., 2017). The pseudo-anonymity afforded by cryptocurrencies facilitates transnational operations, enabling offenders to obscure attribution and evade prosecution. This dynamic aligns with routine activity theory, wherein the proliferation of vulnerable systems – such as IoT-enabled industrial controls – creates abundant crime opportunities in the absence of capable guardianship (Cohen & Felson, 1979).

From a security standpoint, the implications are profound. Cryptomining malware embedded within everyday technologies, particularly, critical infrastructure can degrade system performance, increase latency, and compromise operational resilience, particularly in sectors such as energy, healthcare, and transportation where continuity is paramount. The diversion of computational resources may also mask more destructive payloads, positioning cryptojacking as a potential precursor to sabotage or espionage. Furthermore, the distributed nature of these attacks complicates detection and incident response, necessitating a paradigm shift from device-centric to network-level monitoring and forensic readiness. In this context, cryptomining malware should be approached not merely as an economic nuisance but as a strategic threat vector capable of undermining national security and public safety.

### Limitations

As with any research, this systematic review suffers from a number of limitations that merit attention. A primary limitation centred around the inclusion of grey literature. The grey literature used in this review were mostly governmental and non-governmental reports, as some of these were readily available via Policy Commons and ProQuest. However, we acknowledge there is a very active research field within the industry – including many white papers, industry reports and enterprise blog posts – which was beyond the scope of the review protocol. As there was no systematic method to retrieve all these – given the nature of this review – these were not included in this work. A more comprehensive review including such literature can be conducted; however, such work would not be systematic in nature. Regardless, this work provides a good foundation for future investigations to build upon.

Another key issue was the lack of standardisation of measurements across the literature. Given the multi-disciplinary nature of the research questions, there is no uniform or standard methodology across all considered papers. Measurements (and indeed naming conventions) of cryptomining malware and its impact also vary in the literature; thus, hindering a uniform understanding of its full impact across the years.

## Conclusion

This study systematically screened 897 articles across four major academic databases, with 119 articles included for detailed analysis. Through this comprehensive review, we identified key historical developments, prevailing detection strategies, current security recommendations, and anticipated future directions in the domain of cryptomining malware.

Our findings reveal that cryptomining malware has been propagated through a range of vectors, including browser-based, mobile-based, IoT-based, file-based, and cloud-based mechanisms. Across the reviewed literature, malware detection was a recurring theme, with techniques broadly categorized into static, dynamic, and network-based approaches. Security recommendations largely centered on technical measures, particularly the development of specialized tools and systems designed to detect and prevent cryptojacking. Looking ahead, the consensus within the academic community suggests that cryptomining malware will remain a persistent and increasingly sophisticated threat. As such, continued research, technological innovation, and cross-sector collaboration are essential to mitigating its impact and safeguarding digital infrastructures.

Studying and developing security recommendations in order to reduce cryptomining malware has wide implications. Theft of victims’ computer resources has negative environmental consequences, and the economic impact is of particulat importance considering contemporary concerns over energy availability and cost-of-living increases following the COVID-19 pandemic. Moreover, as cryptojacking is primarily an economically driven crime, it is important to remember that it is only a smaller part subset of a (cyber-)criminal ecosystem. Often, cryptomining malware is diffused in tandem with and via phishing or botnets, two fields which have received extensively coverage in the literature. Furthermore, cryptojacking may serve as a form of (passive) income to multiple malicious actors involved not only in cybercrime but also physical crime, including organised crime and terrorist groups.

It is our hope that in conducting this systematic review the reader gains a clear representation of the literature available on cryptomining malware, which will also be of use to decision makers. This review showed that cryptomining malware covertly exploits computational resources, extends harm across economic and ecological domains and avoid traditional legal thresholds. Its distributed nature and hidden operation demands an holistic approach to curb its potential impact. It is evident from this work that multiple stakeholders will need to on a holistic approach to curb the potential impact of cryptomining malware.

## Data Availability

The dataset used and analysed in the current study are available from the corresponding author on reasonable request.
